# Selective Ion Changes during Spontaneous Mitochondrial Transients in Intact Astrocytes

**DOI:** 10.1371/journal.pone.0028505

**Published:** 2011-12-01

**Authors:** Guillaume Azarias, Jean-Yves Chatton

**Affiliations:** 1 Department of Cell Biology and Morphology, University of Lausanne, Lausanne, Switzerland; 2 Cellular Imaging Facility, University of Lausanne, Lausanne, Switzerland; Université Joseph Fourier, France

## Abstract

The bioenergetic status of cells is tightly regulated by the activity of cytosolic enzymes and mitochondrial ATP production. To adapt their metabolism to cellular energy needs, mitochondria have been shown to exhibit changes in their ionic composition as the result of changes in cytosolic ion concentrations. Individual mitochondria also exhibit spontaneous changes in their electrical potential without altering those of neighboring mitochondria. We recently reported that individual mitochondria of intact astrocytes exhibit spontaneous transient increases in their Na^+^ concentration. Here, we investigated whether the concentration of other ionic species were involved during mitochondrial transients. By combining fluorescence imaging methods, we performed a multiparameter study of spontaneous mitochondrial transients in intact resting astrocytes. We show that mitochondria exhibit coincident changes in their Na^+^ concentration, electrical potential, matrix pH and mitochondrial reactive oxygen species production during a mitochondrial transient without involving detectable changes in their Ca^2+^ concentration. Using widefield and total internal reflection fluorescence imaging, we found evidence for localized transient decreases in the free Mg^2+^ concentration accompanying mitochondrial Na^+^ spikes that could indicate an associated local and transient enrichment in the ATP concentration. Therefore, we propose a sequential model for mitochondrial transients involving a localized ATP microdomain that triggers a Na^+^-mediated mitochondrial depolarization, transiently enhancing the activity of the mitochondrial respiratory chain. Our work provides a model describing ionic changes that could support a bidirectional cytosol-to-mitochondria ionic communication.

## Introduction

Cellular energy metabolism requires a dynamic equilibrium between ATP consumption and ATP production by cytosolic enzymes and mitochondria. To adapt their energy metabolism to constantly changing energy substrate availability and energy demands, transcriptional factors and intracellular ion concentration modulate mitochondrial oxidative metabolism. For example, transcription coactivators such as the peroxisome proliferator-activated receptor γ coactivator-1 family are emerging as central intracellular energy sensors to guarantee metabolic flexibility [1]. Changes in cytosolic ion concentrations have also been shown to link cellular activity and mitochondrial oxidative metabolism. Cytosolic Ca^2+^ concentration rapidly regulate the activity of Ca^2+^-sensitive mitochondrial dehydrogenases, linking cellular Ca^2+^ homeostasis with metabolism [2]. Intracellular pH changes influence the mitochondrial oxidative metabolism in astrocytes [3]. In pathological conditions, the disruption of mitochondrial oxidative metabolism regulation leads to cytotoxic accumulation of free radicals [4] or energy depletion [5], [6].

Although the mitochondrial ionic concentrations have always been thought to be tightly regulated and maintained stable by transport systems [7], the use of mitochondria-targeted fluorescent probes revealed the existence of rapid and reversible alterations of ionic mitochondrial concentrations at the level of single mitochondria. These events, described as spontaneous, as their triggering factor has not been identified, have been first discovered using electrical potential-sensitive mitochondrial dyes. Such mitochondrial transients occur in several cell types including astrocytes [8], neurons [9], cardiomyocytes [10], smooth muscle cells [11] and COS-7 cells [12]. It was recently reported that mitochondria exhibit transient increases of mitochondrial reactive oxygen species (ROS) concentration (“superoxide flashes”) in cultured cells as well as in the myocardium of intact beating hearts [10]. However, the specificity of the probe used in this study is debated, as this type of probe may exhibit sensitivity to ATP concentration [13] and pH [10], [14], [15], raising questions regarding the interpretation of such fluorescence signals.

We recently showed that individual mitochondria in intact astrocytes exhibit spontaneous increase in their Na^+^ concentration that were coincident with mitochondrial depolarization [16]. We presented a body of evidence suggesting that Na^+^ entry is mediated by an electrogenic mitochondrial a cation uniporter and that the mitochondrial Na^+^/H^+^ exchanger contributed to restore mitochondrial Na^+^ concentration to basal level. Finally, our findings suggested that the cellular ATP level modulates the frequency of mitochondrial Na^+^ transient activity. However, the detailed mechanisms mediating Na^+^ entry into mitochondria as well as events triggering mitochondrial Na^+^ transients remain to be clarified.

In the present study, we developed fluorescence imaging methods in intact resting astrocytes to disentangle the dynamics of mitochondrial proton concentration change during a mitochondrial Na^+^ spikes. We found that mitochondrial spikes involve the coincident alteration of Na^+^, mitochondrial electrical potential, proton concentration as well as the mitochondrial ROS (mROS) level without generating detectable alteration of the mitochondrial Ca^2+^ level. Finally, we show that the free cytosolic Mg^2+^ concentration in the immediate vicinity of spiking mitochondria is transiently decreased. Therefore, we propose a model of coincident ionic-specific fluxes between mitochondria and the cytosol that reconciles the body of recent data.

## Results

### Spontaneous alkaline transients in individual mitochondria of intact astrocytes

**Figure 1:**
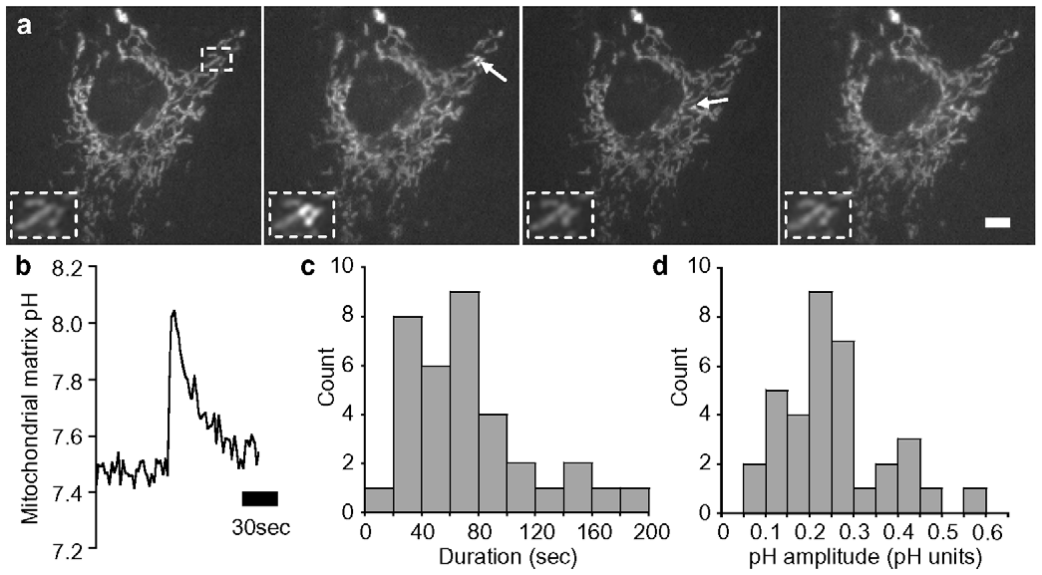
Spontaneous alkaline transients in individual mitochondria. (**a**) Astrocyte mitochondria exhibit individual spontaneous transient alkalinization of mitochondrial matrix as revealed using the mitochondrially-targeted pH-sensitive sensor MitoSypHer. A mitochondrion exhibiting a mitochondrial alkaline transient is indicated with an arrow in the bottom image. Images were taken at t = 3, 19, 55 and 70 sec. Scale bar: 10 µm (**b**) Example trace of calibrated mitochondrial alkaline transient in single mitochondrion of resting astrocytes. (**c**) Distribution of duration of spontaneous mitochondrial alkaline transients. (**d**) Distribution of amplitude of spontaneous mitochondrial alkaline transients. (n = 6 exp, 30 mitochondria)

In intact astrocytes, individual mitochondria have been shown to exhibit spontaneous transient mitochondrial depolarization [8]. In myocytes, spontaneous individual mitochondrial depolarization have been linked to an increase in the activity of the mitochondrial respiratory chain [10]. Both events could have an impact on mitochondrial pH. To directly investigate whether spontaneous transients occur in astrocytes and alter mitochondrial matrix pH, we used transfected astrocytes expressing MitoSypHer, a genetically-encoded fluorescent probe targeted to the mitochondrial matrix and sensitive to pH [3], [17] and monitored the changes of pH in the matrix of individual mitochondria in primary mouse astrocytes. The pattern of MitoSypHer fluorescence in astrocytes was found to be typical of mitochondria with dark nuclei and rod-like structures which colocalized with mitochondrial stains, as described previously [3]. MitoSypHer excitation ratio 490 nm/420 nm enabled monitoring mitochondrial matrix pH in real time by fluorescence microscopy. **Figure 1a** depicts an image sequence and shows individual mitochondria spontaneously lighting up (see also **Movie S1**; provided in supplementary material). Similar to mitochondrial Na^+^ transients [16], individual mitochondria usually showed 1–2 transients in 2-min periods of recording (**Figure 1b**). The analysis of fluorescence changes in individual mitochondria indicated that the duration of transients was found to be 71±7sec (median: 61sec; **Figure 1c**). The observed MitoSypher spontaneous transients always displayed a positive deflection, corresponding to alkalinization. Calibration of mitochondrial pH indicated that the basal mitochondrial matrix pH was 7.44±0.02 pH units and the amplitude of transients averaged 0.24±0.02 pH units (median: 0.23 pH units; **Figure 1d**). The rate of H^+^ concentration changes during transients was estimated at 158±24 nM·sec^−1^ and 25±5 nM·sec^−1^ for influx and efflux, respectively. In this report, we refer to MitoSypHer transients in individual mitochondria as "mitochondrial alkaline transients".

### Pattern of ionic changes during spontaneous mitochondrial alkaline transients

**Figure 2:**
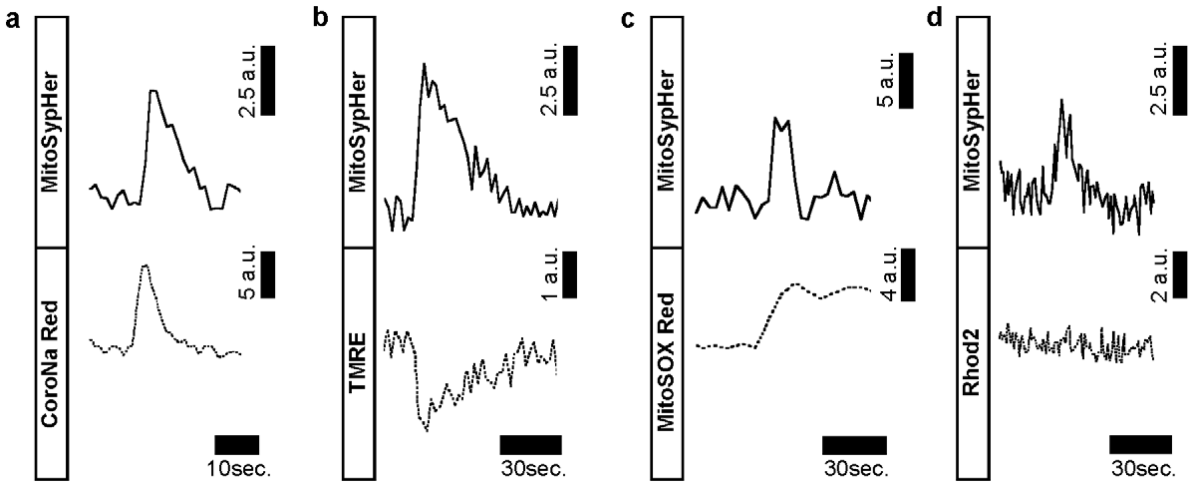
Spontaneous mitochondrial alkaline transients are coincident with mitochondrial Na^+^ transients, mitochondrial depolarization, and bursts of superoxide generation. (**a**) Mitochondrial alkaline transients are coincident with mitochondrial Na^+^ transients. Astrocytes were transfected with MitoSypHer and subsequently loaded with CoroNa Red to monitor pH and Na^+^, respectively in the same mitochondria. Experimental trace depicting spontaneous pH changes (top trace) and Na^+^ changes (bottom trace). (n = 6 exp, 27 mitochondria). (**b**) Mitochondrial alkaline transients are coincident with mitochondrial depolarization. MitoSypHer transfected astrocytes were loaded with TMRE in unquenched mode to monitor pH and electrical potential, respectively in the same mitochondria. Experimental trace depicting spontaneous pH changes (top trace) and mitochondrial electrical potential changes (bottom trace). (n = 6 exp, 14 mitochondria) (**c**) Mitochondrial alkaline transients are coincident with burst of superoxide generation. MitoSypHer transfected astrocytes were loaded with MitoSOX Red to monitor pH and mROS level, respectively in the same mitochondria. Experimental trace depicting spontaneous pH changes (top trace) and mROS (bottom trace). (n = 8 exp, 24 mitochondria). (**d**) Mitochondrial alkaline transients are not coincident with detectable changes in mitochondrial Ca^2+^ concentration. MitoSypHer transfected astrocytes were labeled with Rhod2 to monitor pH and Ca^2+^ in the same mitochondria. Experimental trace depicting spontaneous pH changes (top trace) and Ca^2+^ changes changes (bottom trace). (n = 7 exp, 16 mitochondria).

As was observed with mitochondrial Na^+^ transients [16], mitochondrial alkaline transients occurred spontaneously in individual mitochondria and had durations in the same order of magnitude as mitochondrial Na^+^ transients. Therefore, we tested if spontaneous mitochondrial Na^+^ transients were temporally and spatially correlated with mitochondrial alkaline transients by loading MitoSypHer transfected astrocytes with the mitochondrial selective Na^+^ indicator CoroNa Red [16], [18] to monitor both Na^+^_mit_ and pH_mit_ in the same mitochondrion. As seen in **Figure 2a**, we observed that spontaneous mitochondrial alkaline transients coincided with mitochondrial Na^+^ transients. The pH and Na^+^ transient alterations followed a similar time course (**Figure S1)** that on average did not show significant difference in duration (p = 0.49 using Student t-test).

As we previously reported that mitochondrial Na^+^ transients were accompanied with mitochondrial depolarizations [16], we tested whether the same was true for mitochondrial alkaline transients. We addressed this issue by loading MitoSypHer transfected astrocytes with the mitochondrial potential sensitive dye tetramethylrhodamine ethyl ester (TMRE) used at low concentrations (10 nM). Uncoupling mitochondria using p-trifluoromethoxy carbonyl cyanide phenyl hydrazone (FCCP) led to a decrease in both fluorescence of MitoSypHer and TMRE indicating that TMRE was being used in unquenched mode (**Figure S2**). **Figure 2b** shows that spontaneous pH transients coincided with transient mitochondrial depolarizations. Taken together, these results indicate that during a mitochondrial spike, Na^+^ and proton concentrations evolved in an opposite manner, and that the mitochondrial electrical potential transiently depolarized.

In a next phase, we investigated whether the mitochondrial alkaline transients were related with changes in the mitochondrial respiratory chain activity. We chose to monitor the production of mROS that represent natural byproducts of the mitochondrial respiratory chain [4]. For this purpose, we used the mitochondrial specific fluorescent probe MitoSOX Red which becomes fluorescent upon oxidation by mitochondrial superoxide production [19]. In these experiments, we used Antimycin A, an inhibitor of the complex I of the mitochondrial respiratory chain as positive control of the reactivity of the probe. Antimycin A led simultaneously to a decrease in MitoSypHer fluorescence indicating a mitochondrial matrix acidification and an increase in MitoSOX Red fluorescence (**Figure S3**), as expected for an increase in ROS production [4], [20]. Among 26 analyzed transients, 18 alkaline transients (69%) were accompanied with a significant increase in MitoSOX Red consistent with a transient increase in mROS production (**Figure 2c**). During an alkaline transient, the MitoSOX Red fluorescence increased in a step-like fashion by 11.9±0.2% of the basal MitoSOX Red fluorescence, Antimycin A increased the fluorescence by 31.4±4.0% the basal fluorescence.

As astrocytes may experience fast intracellular Ca^2+^ elevations during stimulations with various agonists (see e.g. [21]), we asked whether mitochondrial transients involved other ions such as Ca^2+^. We tested this hypothesis by monitoring mitochondrial alkaline transients and mitochondrial Ca^2+^ concentration in the same astrocytes using the Ca^2+^-sensitive mitochondrial probe Rhod-2 AM, as performed in previous studies [20]. Mitochondrial depolarization caused by FCCP led to a drop in both Rhod-2 and MitoSypHer fluorescence (**Figure S4**). This observation confirmed that mitochondrial matrix pH and Ca^2+^ are kept notably higher than in the cytosol before FCCP uncoupling, due to the activity of the mitochondrial respiratory chain. In these experiments, we did not find detectable alterations of mitochondrial Ca^2+^ level during mitochondrial alkaline transients (**Figure 2d**). It is noteworthy that in several experiments, the mitochondrial Ca^2+^ level spontaneously displayed slow oscillations in the entire population of mitochondria at a stable frequency (*not shown*). These Ca^2+^ oscillations in the mitochondria population, likely reflecting whole-cell cytosolic Ca^2+^ oscillations, were without temporal or spatial coincidence with mitochondrial alkaline transients. Consistent with this observation, we found that evoking Ca^2+^ responses using the agonist of metabotropic glutamate receptors (RS)-3,5-dihydrophenylglycine (DHPG) did not alter the probability of mitochondrial Na^+^ transients appearance (control: 478±234 transients/min, DHPG 100 µM: 489±261, transients/min, DHPG 1 mM: 484±284 transients/min, p>0.05 using Student t-test against control, n = 8 exp). Thus, these results indicated that mitochondrial transients correspond to changes in pH and Na^+^ concentration—but not in Ca^2+^ concentration—within the same mitochondrion.

### Spontaneous changes in free magnesium concentration accompanied transients in individual mitochondria

**Figure 3:**
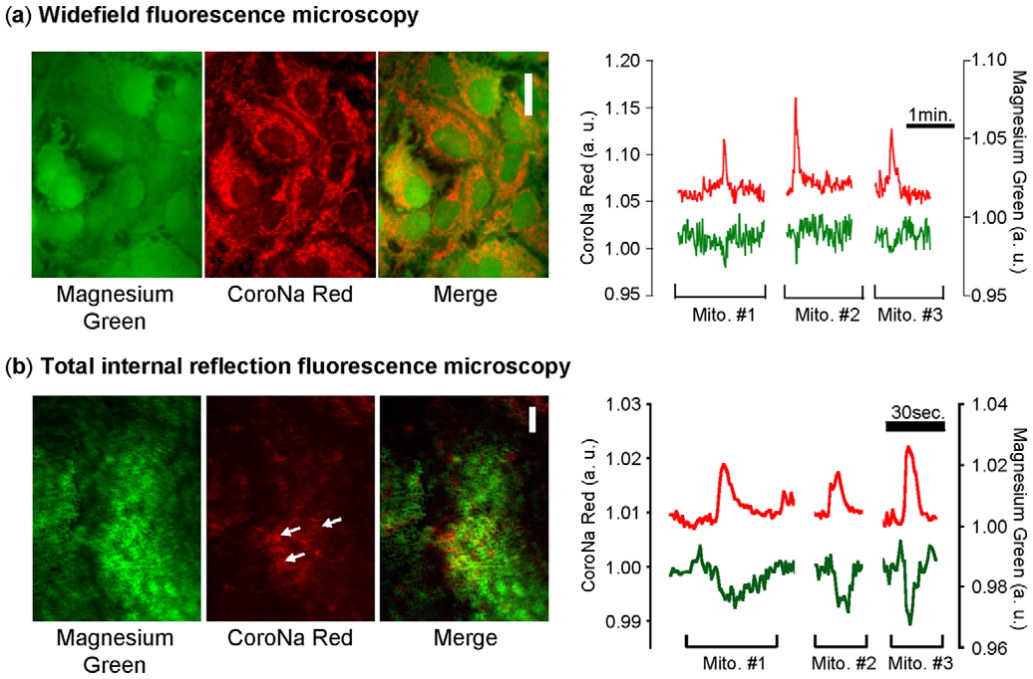
Mitochondrial Na^+^ transients are coincident with transient decrease in cytosolic Mg^2+^ concentration. Astrocytes were loaded with Magnesium Green AM and CoroNa Red to monitor local free Mg^2+^ concentration and mitochondrial Na^+^ spikes. (**a**) Fluorescence images of astrocytes showing the typical cytosolic and mitochondrial patterns of Magnesium green and CoroNa Red, respectively observed under widefield microscopy. Experimental trace depicting spontaneous mitochondrial Na^+^ spikes (red trace, left axis) and free Mg^2+^ concentration (green trace, right axis). Scale bar: 20 µm (n = 6 exp, 24 mitochondria). (**b**) Fluorescence images of an astrocyte showing the fluorescence patterns of Magnesium green and CoroNa Red, respectively observed under TIRF microscopy. White arrows indicate where mitochondria are almost entirely located in the field of the evanescent wave. Experimental trace depicting spontaneous mitochondrial Na^+^ spikes (red trace, left axis) and free Mg^2+^ concentration (green trace, right axis). Scale bar 10 µm (n = 5 exp, 19 mitochondria).

The mechanisms leading to the initiation of a mitochondrial Na^+^ transient in astrocytes have not been firmly identified [16]. However, we found evidence that the frequency of mitochondrial transients was correlated with the global cellular ATP level; we therefore investigated the possibility of a link between mitochondrial Na^+^ transients and ATP levels in the local mitochondrial microenvironment. Using widefield fluorescence microscopy, we simultaneously monitored mitochondrial Na^+^ and cytosolic free Mg^2+^ concentration using the fluorescent indicators CoroNa Red and Magnesium Green, respectively. The punctate CoroNa Red fluorescent pattern was typical of mitochondrial staining [3], [16], whereas the Magnesium Green fluorescence pattern was typical of a cytosolic probe, with a homogeneous cytosolic fluorescence and a bright nucleus (**Figure 3a**). As previously described [22], [23], [24], the Magnesium Green fluorescent signal can be used to detect changes in ATP concentration. While an increase in ATP hydrolysis, which releases a substantial amount of Mg^2+^, causes an increase in Magnesium Green fluorescence intensity, an increase in ATP concentration can be detected by a decrease in Magnesium Green fluorescence. In our experiments, the mitochondrial uncoupler FCCP was used as a control to maximally deplete cellular ATP and led to an increase in the Magnesium Green fluorescence intensity (**Figure S5**). **Figure 3a** shows that mitochondrial Na^+^ transients were accompanied with a simultaneous transient decrease of free Mg^2+^ concentration as monitored by widefield microscopy. In order to refine the spatial discrimination of the measurement and obtain a more localized measurement of the cytosolic free Mg^2+^ concentration, we used the same experimental protocol using total internal reflection fluorescence (TIRF) microscopy. This imaging method enables monitoring the fluorescence signal in a ∼100 nm-thick optical section below the plasma membrane. **Figure 3b** shows that this imaging modality confirmed that mitochondrial Na^+^ spikes were accompanied with a transient decrease in the free Mg^2+^ concentration, suggesting the occurrence of a brief increase of ATP availability in the immediate vicinity of mitochondria exhibiting a Na^+^ transient.

## Discussion

This study demonstrates that mitochondria in primary astrocytes, in the absence of external stimuli, spontaneously display rapid transients that engage transmembrane movement of several ionic species, along with electrical potential. We found that mitochondria exhibiting transients experienced opposite changes in Na^+^ and H^+^ concentrations, whereas the Ca^2+^ level remained stable. In addition we show evidence suggesting that a microdomain of decreased free Mg^2+^ concentration, potentially associated with increased ATP concentration, was coincident with mitochondrial Na^+^ transient.

Despite the fact that astrocytes are considered as having a mainly glycolytic metabolism, they contain a high density of mitochondria in their entire cytoplasm. Using several fluorescent probes targeted to mitochondria and sensitive to mitochondrial electrical potential, Na^+^ concentration or pH, we observed that individual mitochondria display transient alterations of their ionic content whereas nearby mitochondria remained stable. In cultured astrocytes, mitochondria appear to behave as functionally distinct entities as mitochondrial Na^+^ concentration changes were observed in individual mitochondria without transmission to neighbors [16], which was also observed with mitochondrial pH transients in the present study. The same conclusion was reached for other cell types such as hepatocytes [25], cardiac myocytes [10], [26], or hippocampal neurons [10]. This contrasts with certain cell types, such as HeLa cells [27], where mitochondria are organized as an interconnected network. Mitochondrial movement was found to be negligible in the time frame of our experiments and no obvious correlation was found between mitochondrial transients and rare mitochondrial movements. Mitochondria may also undergo fission and fusion in living cells for instance to allow them to reach domains where ATP synthesis is needed [28]; however, as was the case for mitochondrial movement, we did not find evidence of such events in the time scale of the described pH or Na^+^ transients.

We previously proposed that mitochondrial Na^+^ transients are mediated by the entry of Na^+^ through an electrogenic cation uniporter [16] and that mitochondrial Na^+^ concentration was restored to basal level by the activity of the mitochondrial Na^+^/H^+^ exchanger. Extruding Na^+^ through Na^+^/H^+^ exchange would be expected to lead to matrix acidification. However, in the present experiments, the observed pH transients were always alkaline and no acidic transient were found to occur, which contrasts with the decrease in matrix pH seen in the mitochondrial population seen upon stimulation with the neurotransmitter glutamate [3]. If the Na^+^/H^+^ exchanger is likely not responsible for the upstroke of the transient, it could nevertheless be involved in the recovery phase. This result suggests that mechanisms more complex than only Na^+^/H^+^ exchanger are involved during a transient. In a model of isolated cardiac mitochondria, transient changes in electrical potential was proposed to involve the pH-mediated opening of a H^+^ channel [29]. If a similar mechanism prevailed in astrocytes with the opening a H^+^-selective channel, it would be expected to cause transient drops in matrix pH instead of the observed alkalinization.

Our mutiparameter analysis indicated that mitochondrial alkaline transients were found to coincide with mitochondrial depolarizations. Mitochondrial alkaline transients could therefore arise from the opening of an electrogenic cation uniporter, causing mitochondrial depolarization. This depolarization is expected to enhance the mitochondrial electron transfer chain and accelerate the extrusion of protons outside of mitochondrial matrix, causing the alkalinization of the matrix. The existence of the transient increase in mitochondrial respiratory chain activity is supported by the transient increase in superoxide production observed in the present study, which was also found in myocytes [10] or skeletal muscle mitochondria [20], that were both shown to be concomitant with mitochondrial depolarization.

We reported that thapsigargin, a non competitive inhibitor of the SERCA inducing the release of Ca^2+^ from the endoplasmic reticulum, did not alter mitochondrial Na^+^ transient activity [16]. Consistent with this observation, astrocytes expressing MitoSypHer and labeled with the mitochondrially targeted Ca^2+^-sensitive dye Rhod-2 exhibited spontaneous pH transients without coincident alteration of Rhod-2 fluorescence. In addition, the frequency of repetitive mitochondrial depolarization was found to be not affected by the Ca^2+^ level surrounding isolated mitochondria [29]. Finally, mitochondrial superoxide transients were also found not to be accompanied by mitochondrial Ca^2+^ spiking [10], [20]. These data exclude the spontaneous Ca^2+^ release from endoplasmic reticulum as an obligatory mechanism to trigger mitochondrial transients.

The several recent studies reporting spontaneous mitochondrial transients of electrical potential, Na^+^, superoxide (see *e.g.* [8], [9], [10], [11], [12], [20], [29]) could not identify a triggering mechanism. In a previous study [16], we had identified a potential link between the cellular ATP level and the frequency of mitochondrial Na^+^ transients. By using high resolution measurements of free Mg^2+^ to indirectly monitor ATP [23], we found that the free Mg^2+^ concentration decreased in the vicinity of a mitochondrion exhibiting a transient with the same kinetics. Because of the significant Mg^2+^-chelating action of ATP, a decrease in free Mg^2+^ concentration is a strong evidence for an increase in ATP concentration. The existence of ATP microdomains have been reported in pancreatic islet beta-cells [30] and might also exist in other cell types [26]. This new result could indicate that microdomains of ATP around mitochondria induce individual mitochondrial spiking, as was suggested recently [13]. The spatial resolution of the imaging setup used for monitoring transient ATP domains was not sufficient to distinguish if the origin of the ATP concentration changes was cytosolic or mitochondrial. An attractive possibility would be that the ATP transients are generated by mitochondria because mitochondrial transients are accompanied with mitochondrial alkaline transients, increasing the chemical gradient of proton for mitochondrial ATP synthesis. However, two elements suggest that it is not the case: the mitochondrial electrical potential, which is the major driving force for the ATP synthase, is decreased during mitochondrial transients; the blocker of the ATP synthase oligomycin (1 µM) did not prevent transient decreases in free magnesium (not shown). One could also postulate that free Mg^2+^ could itself play a role in the triggering of transients. Indeed, a Na^+^-permissive mitochondrial cation uniporter was described to be opened in low divalent cation containing medium and with its open-state induced by ATP [7].

**Figure 4:**
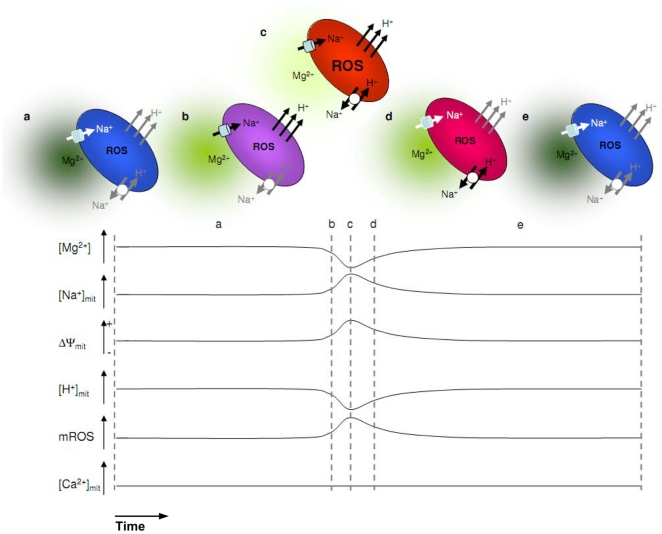
Model for ionic alterations occurring during mitochondrial transients. (**a**) and (**e**) are resting mitochondria in the cytosol with a basal free Mg^2+^ level. (**b**) to (**d**) illustrate the putative different states of the same mitochondrion experiencing a spontaneous transient, according to the proposed model. The dark to white green squares near the mitochondrion illustrate the normal resting to low Mg^2+^ concentration, respectively. The cold blue to hot red colors of the mitochondria illustrate the mitochondrial electrical potential, from hyperpolarized to depolarized, respectively. Na^+^ and H^+^ pathways are represented in white, gray and black corresponding to inactive, constitutive and maximal activities, respectively. The bottom traces summarize the respective changes in Mg^2+^ as well as mitochondrial Na^+^, electrical potential, H^+^, ROS, and Ca^2+^, measured in the present study.

Studies reporting various spontaneous mitochondrial transients have many common features [8], [9], [10], [11], [12], [20], [29]; however, not only could they not identify the mechanism triggering the events, they also could not assign functional roles to them. The mutiparameter analysis presented in this study, allows us to propose a sequence of events occurring during mitochondrial transients in astrocytes, as depicted in **Figure 4**. Our measurements suggest that rapid localized ATP changes decreases the free Mg^2+^ level, activating the electrogenic and Na^+^-permeable mitochondrial cation uniporter (**Figure 4b**). The associated mitochondrial depolarization enhances the electron transfer chain and the extrusion of protons across the inner membrane causing a rapid mitochondrial pH rise. The increase in mitochondrial Na^+^ concentration and mitochondrial alkalinization enhance the activity of the mitochondrial Na^+^/H^+^ exchanger (**Figure 4c**). Finally, the closure of the mitochondrial cation uniporter relieves the mitochondrial depolarization, whereas the mitochondrial Na^+^/H^+^ exchanger restores Na^+^ and H^+^ concentrations to basal levels (**Figure 4d**). This model is admittedly speculative inasmuch as the temporal resolution reached by the methods used in our studies did not allow us to define the sequence by which events occurred during the transients. However, it points to the notion that mitochondrial spiking participates in a dynamic process of regulation of mitochondrial energy metabolism at the local level inside living cells. This rapid modulation may encompass, or link, both the energy output of mitochondria and their superoxide generating activity.

## Materials and Methods

### Cell Culture and Solutions

All experimental procedures were carried out according to the Swiss Ordinance on Animal Experimentation and were specifically approved for this study (authorization number 1288.4-5). Cortical astrocytes in primary culture were prepared from 1–3 days-old from C57 Bl 6 mice as described elsewhere [31]. Astrocytes were plated on 20 mm glass coverslips and cultured for 2–4 weeks in DME medium (Sigma) plus 10% FCS.

Experimental solutions contained (mM): NaCl 160, KCl 5.4, HEPES 20, CaCl_2_ 1.3, MgSO_4_ 0.8, NaH_2_PO_4_ 0.78, glucose 5 (pH 7.4) and was bubbled with air. Solutions for dye-loading contained (mM): NaCl 160, KCl 5.4, HEPES 20, CaCl_2_ 1.3, MgSO_4_ 0.8, NaH_2_PO_4_ 0.78, glucose 20 and was supplemented with 0.1% Pluronic F127 (Molecular Probes, Eugene, OR). Calibration solutions for mitochondrial matrix pH contained (mM): NaCl 20, KCl 125, MgCl_2_ 0.5, EGTA 0.2, HEPES 20.

### Astrocyte Transfection

Two-week old astrocytes were placed in 2 ml of antibiotic-free and serum-free DMEM medium with Fugene (Roche) and DNA encoding for MitoSypHer with a ratio of 2 µg DNA for 12 µL FuGene. After four hours, the medium was replaced with DME medium plus 10% serum and cells were used 2–3 days after transfection for pure astrocyte culture.

### Live Cell Imaging

**Epifluorescence microscopy.** Low-light level fluorescence imaging was performed on an inverted epifluorescence microscope (Axiovert 100 M, Carl Zeiss) using a 40 X 1.3 N.A. oil-immersion objective lens. Fluorescence excitation wavelengths were selected using a monochromator (Till Photonics, Planegg, Germany) and fluorescence was detected using a 12-bit cooled CCD camera (Princeton Instruments). Image acquisition and time series were computer-controlled using the software Metafluor (Molecular Devices) running on a Pentium computer. Cells were loaded in a HEPES-buffered balanced solution and then placed in a thermostated chamber designed for rapid exchange of perfusion solutions [32] and superfused at 35°C. To avoid phototoxicity, excitation intensity was reduced to ∼10 µW (as measured at the entrance pupil of the objective) by means of neutral density filters. For mitochondrial matrix pH measurement, MitoSypHer fluorescence was sequentially excited at 490 and 420 nm and detected at >515 nm. At the end of each experiment, *in situ* calibration was performed as described in [3], [17]. Mitochondrial Na^+^ spiking was investigated as described in [16]. Magnesium Green AM, Rhod-2 and TMRE were used at the concentrations of 10 µM, 2 µM and 10 nM, respectively.

#### Confocal microscopy

Monitoring of mitochondrial matrix pH and mROS production was performed on a TCS SP5 AOBS confocal microscope (Leica Microsystems). MitoSypHer transfected astrocytes were loaded for 17 min with MitoSOX Red (500 nM). MitoSypHer fluorescence was excited using the 488 nm low intensity laser light illumination and fluorescence was collected in the channel 500–530 nm. MitoSOX Red fluorescence was excited using the 514 nm laser light and fluorescence was collected 580–695 nm. As a control, 30 µL Antimycin A (200 µg.mL^−1^) were added to the 270 µL of Hepes-glucose medium at the end of each experiment.

#### TIRF microscopy

The expanded beam of a 488/568 nm argon/krypton multiline laser (20 mW; Laserphysics) passed through an acousto-optic tunable filter laser wavelength selector (VisiTech International) synchronized with a CoolSnap HQ charge-coupled device camera (Roper Scientific) under Metafluor software (Universal Imaging) control and was introduced to the coverslip from the high numerical aperture objective lens (α-plan FLUAR 100X; Zeiss). Light entered the coverslip and underwent total internal reflection at the glass-cell interface. Simultaneous monitoring of free Mg^2+^ concentration and mitochondrial Na^+^ spikes was assessed using TIRF microscopy using the laser 488 nm and 568 nm for the excitation of Magnesium Green and CoroNa Red, respectively.

#### Selection of the regions of interest

Regions of interests drawn around mitochondria in fluorescence images were selected for analysis if each of the following criteria were fulfilled: (i) the fluorescence pattern of the mitochondrial probe(s) was clearly restricted into mitochondria-like structures and the fluorescence pattern of cytosolic probe was homogeneous; (ii) the baseline signal for fluorescent probes before and after spontaneous mitochondrial transient(s) was stable; (iii) the drug used as a control of probe sensitivity induced a clear or statistically significant alteration of the fluorescence intensity; (iv) the signal-to-noise ratio allowed to clearly identify a spontaneous alteration of the fluorescence intensity before the addition of the drug used as control.

### Statistics

A paired Student's *t* test was performed for each experiment group to assess the statistical significance against respective controls and *, ** and *** refer to *p* values <0.05, 0.01, and 0.001, respectively.

### Materials

All fluorescent probes were from Invitrogen-Molecular Probes. All substances were from Sigma.

## Supporting Information

Figure S1 Mitochondrial alkaline transients are coincident with mitochondrial Na^+^ transients. Astrocytes were transfected with MitoSypHer and subsequently loaded with CoroNa Red to monitor pH and Na^+^, respectively in the same mitochondria. As a control, a drop (30 µL) of the mitochondrial uncoupler FCCP (10 µM) was added to the 270 µL of buffer at the end of each experiment. Representative trace of 6 experiments (27 mitochondria).
(TIF)

Figure S2 Mitochondrial alkaline transients are coincident with mitochondrial depolarization. Astrocytes were transfected with MitoSypHer and subsequently loaded with TMRE to monitor pH and electrical potential, respectively, in the same mitochondria. As a control, the mitochondrial uncoupler FCCP (10 µM, 30 µL) was added to the 270 µL of buffer at the end of each experiment. Representative trace of 6 experiments (14 mitochondria).
(TIF)

Figure S3 Mitochondrial alkaline transients are accompanied with burst of superoxide generation. Astrocytes were transfected with MitoSypHer and subsequently loaded with MitoSOX Red to monitor pH and free radical production, respectively, in the same mitochondria. MitoSOX Red becomes fluorescent upon binding to a free radical. As a control, a drop (30 µL) of antimycin A (200 µg.mL-1) was added to the 270 µL of buffer at the end of each experiment. Representative trace of 8 experiments (24 mitochondria).
(TIF)

Figure S4 Mitochondrial alkaline transients are not coincident with detectable changes in mitochondrial Ca^2+^ concentration. Astrocytes were transfected with MitoSypHer and subsequently loaded with Rhod2 to monitor pH and Ca^2+^ level, respectively in the same mitochondria. As a control, a drop (30 µL) of the mitochondrial uncoupler FCCP (10 µM) was added to the 270 µL of buffer at the end of each experiment. Representative trace of 6 experiments (14 mitochondria). Representative trace of 7 experiments (16 mitochondria).
(TIF)

Figure S5 Mitochondrial Na^+^ transients are coincident with transient decrease in cytosolic free Mg^2+^ concentration. Astrocytes were simultaneously loaded with Magnesium Green and CoroNa Red to monitor the Mg^2+^ concentration and mitochondrial Na^+^ concentration, respectively. As a control, the mitochondrial uncoupler FCCP (10 µM, 30 µL) was added to the 270 µL of buffer at the end of each experiment. Representative trace of 6 experiments (24 mitochondria) and 5 experiments (19 mitochondria) under widefield and TIRF microscopy, respectively.
(TIF)

Movie S1 Movie of spontaneous mitochondrial alkaline transients observed using a widefield fluorescence microscope).
(AVI)
