# Supplementary figures and images for: Selective Ion Changes during Spontaneous Mitochondrial Transients in Intact Astrocytes
Source: PLoS One. 2011 Dec 1;6(12):e28505. doi: 10.1371/journal.pone.0028505 (PMC3228761; doi:10.1371/journal.pone.0028505)

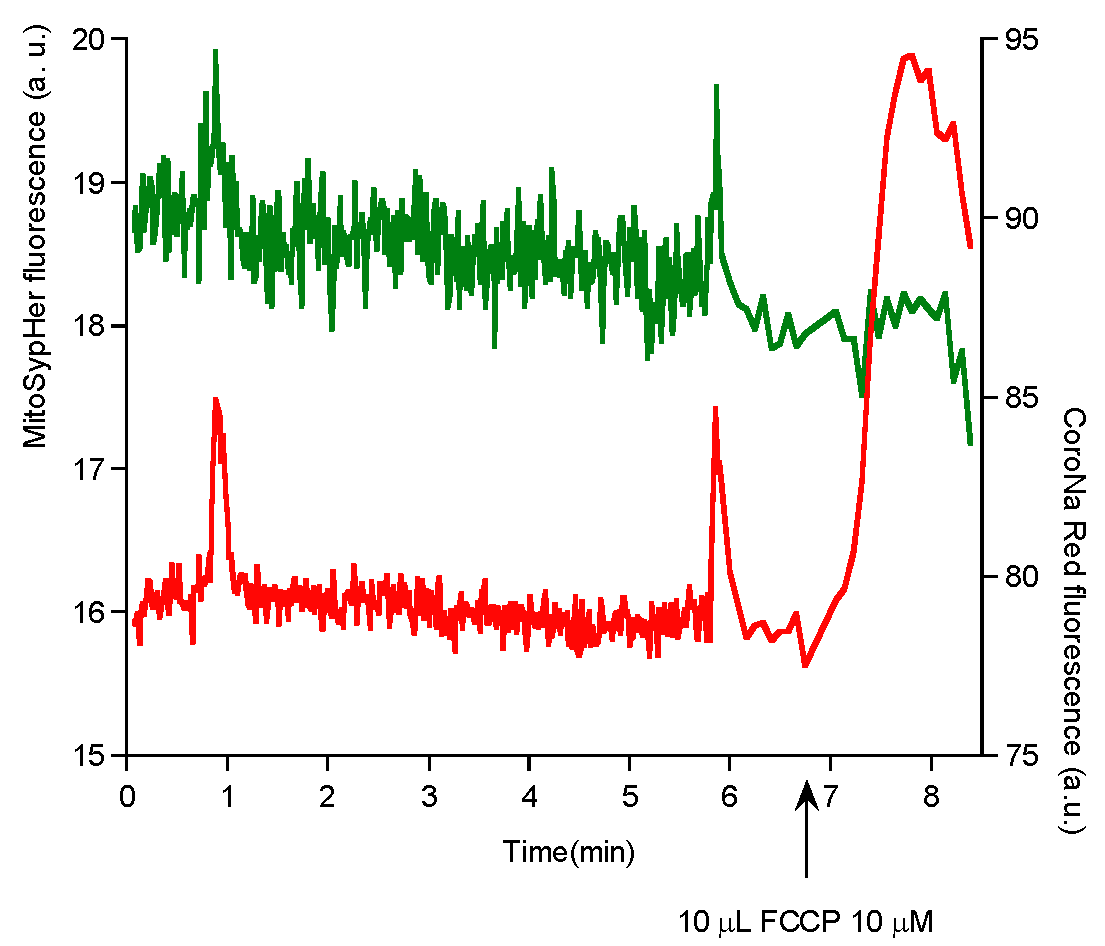

Supplement: Figure S1 — Mitochondrial alkaline transients are coincident with mitochondrial Na+ transients. Astrocytes were transfected with MitoSypHer and subsequently loaded with CoroNa Red to monitor pH and Na+, respectively in the same mitochondria. As a control, a drop (30 µL) of the mitochondrial uncoupler FCCP (10 µM) was added to the 270 µL of buffer at the end of each experiment. Representative trace of 6 experiments (27 mitochondria). (TIF) [file pone.0028505.s001.tif]

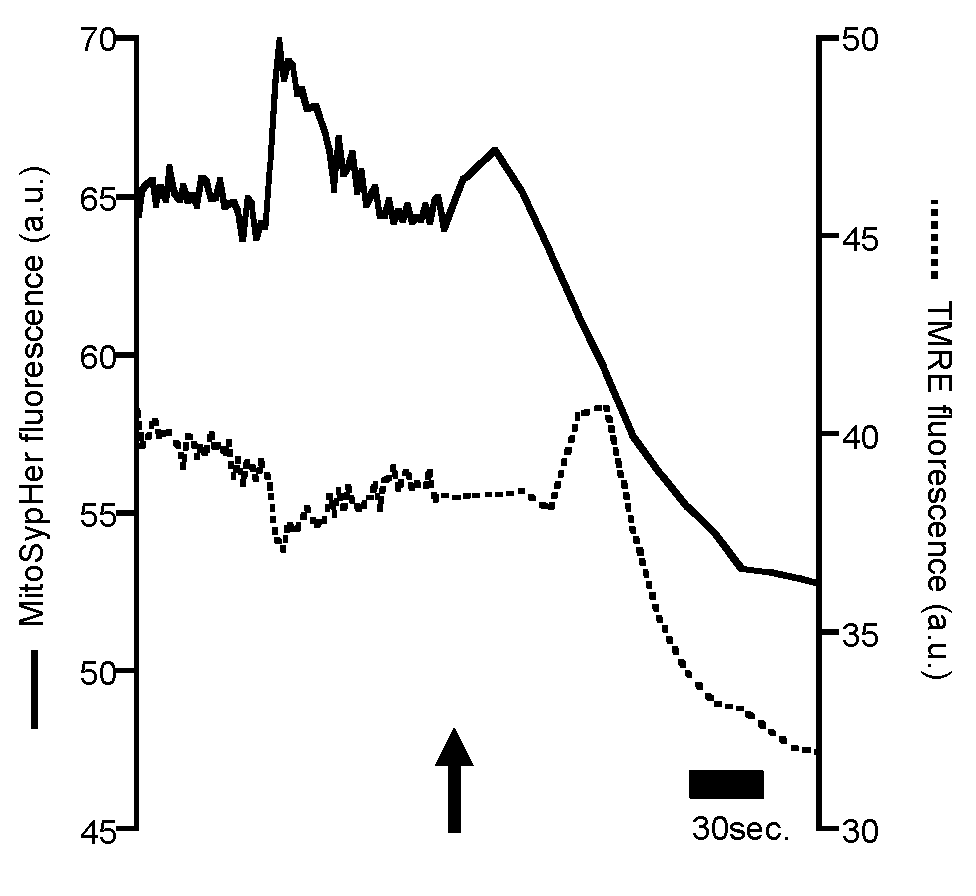

Supplement: Figure S2 — Mitochondrial alkaline transients are coincident with mitochondrial depolarization. Astrocytes were transfected with MitoSypHer and subsequently loaded with TMRE to monitor pH and electrical potential, respectively, in the same mitochondria. As a control, the mitochondrial uncoupler FCCP (10 µM, 30 µL) was added to the 270 µL of buffer at the end of each experiment. Representative trace of 6 experiments (14 mitochondria). (TIF) [file pone.0028505.s002.tif]

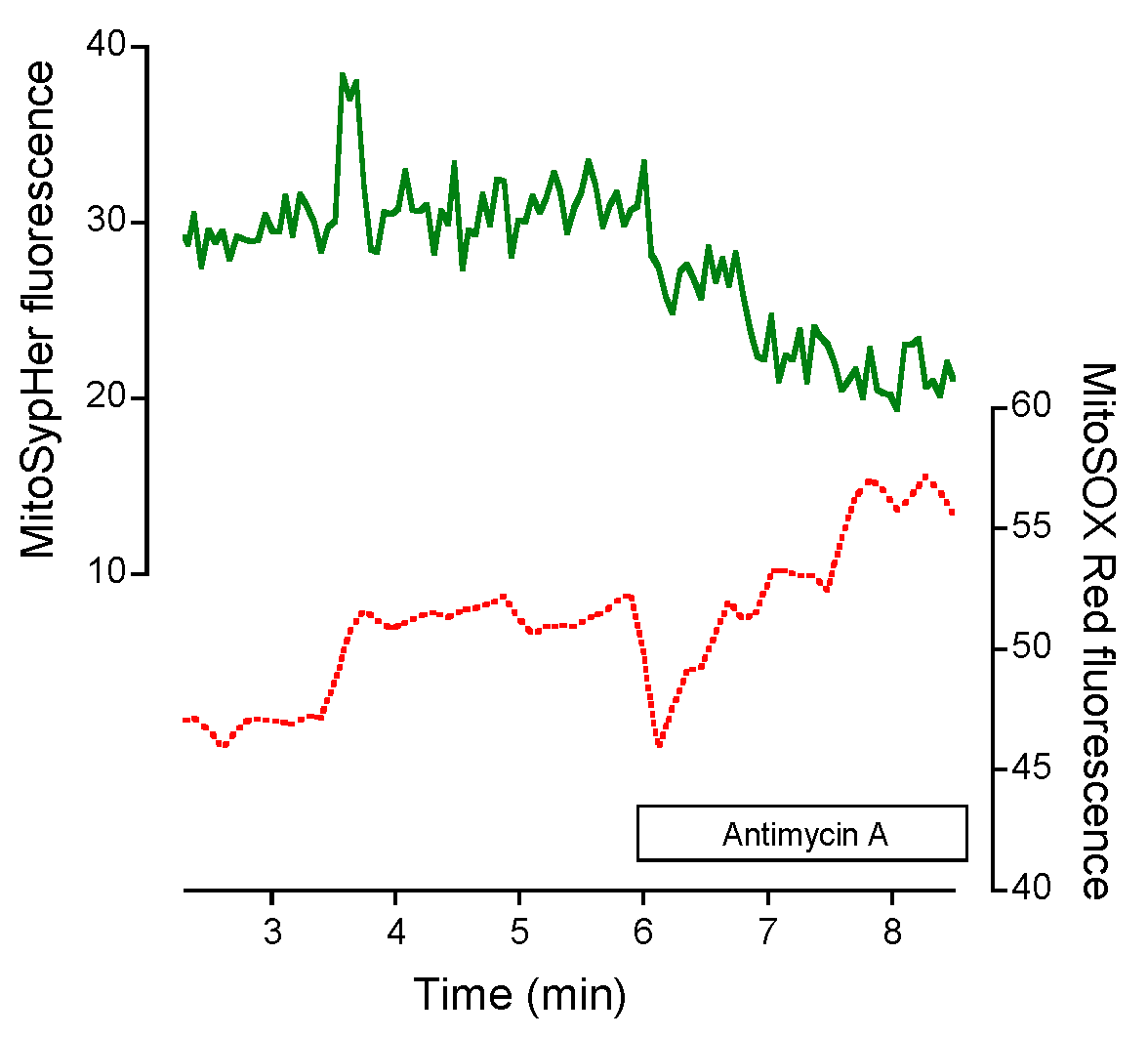

Supplement: Figure S3 — Mitochondrial alkaline transients are accompanied with burst of superoxide generation. Astrocytes were transfected with MitoSypHer and subsequently loaded with MitoSOX Red to monitor pH and free radical production, respectively, in the same mitochondria. MitoSOX Red becomes fluorescent upon binding to a free radical. As a control, a drop (30 µL) of antimycin A (200 µg.mL-1) was added to the 270 µL of buffer at the end of each experiment. Representative trace of 8 experiments (24 mitochondria). (TIF) [file pone.0028505.s003.tif]

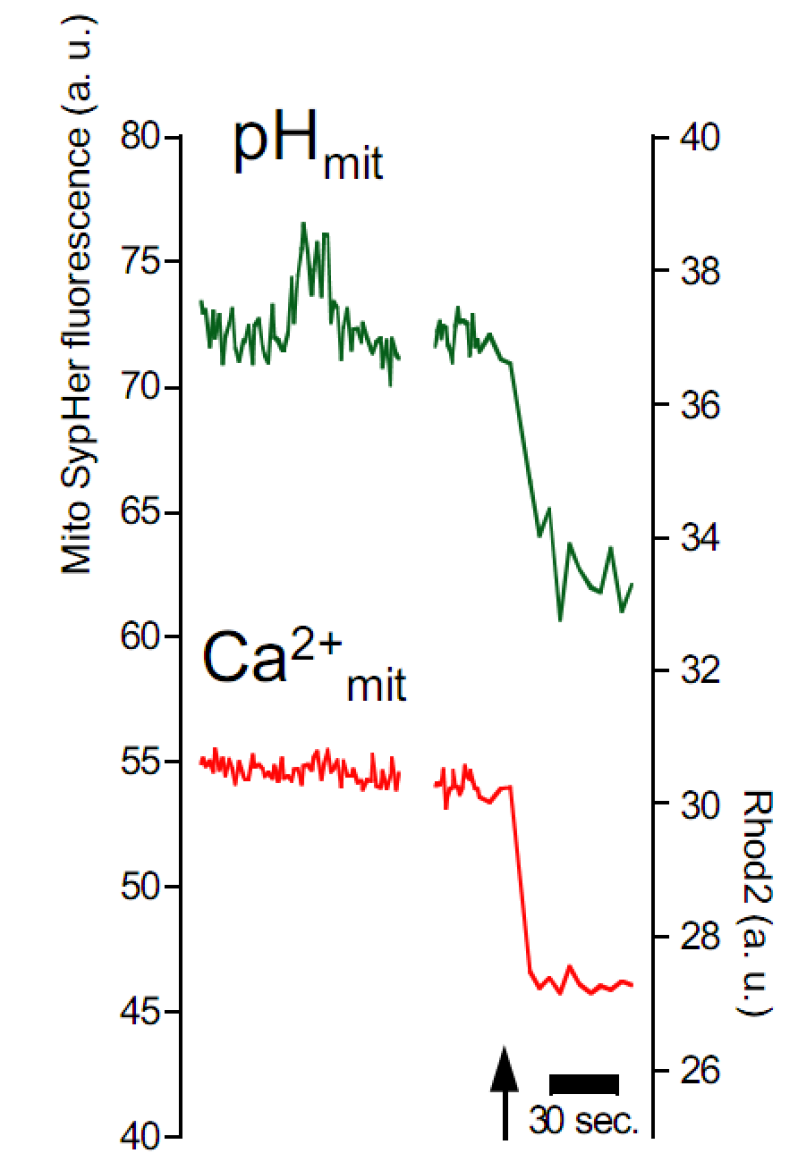

Supplement: Figure S4 — Mitochondrial alkaline transients are not coincident with detectable changes in mitochondrial Ca2+ concentration. Astrocytes were transfected with MitoSypHer and subsequently loaded with Rhod2 to monitor pH and Ca2+ level, respectively in the same mitochondria. As a control, a drop (30 µL) of the mitochondrial uncoupler FCCP (10 µM) was added to the 270 µL of buffer at the end of each experiment. Representative trace of 6 experiments (14 mitochondria). Representative trace of 7 experiments (16 mitochondria). (TIF) [file pone.0028505.s004.tif]

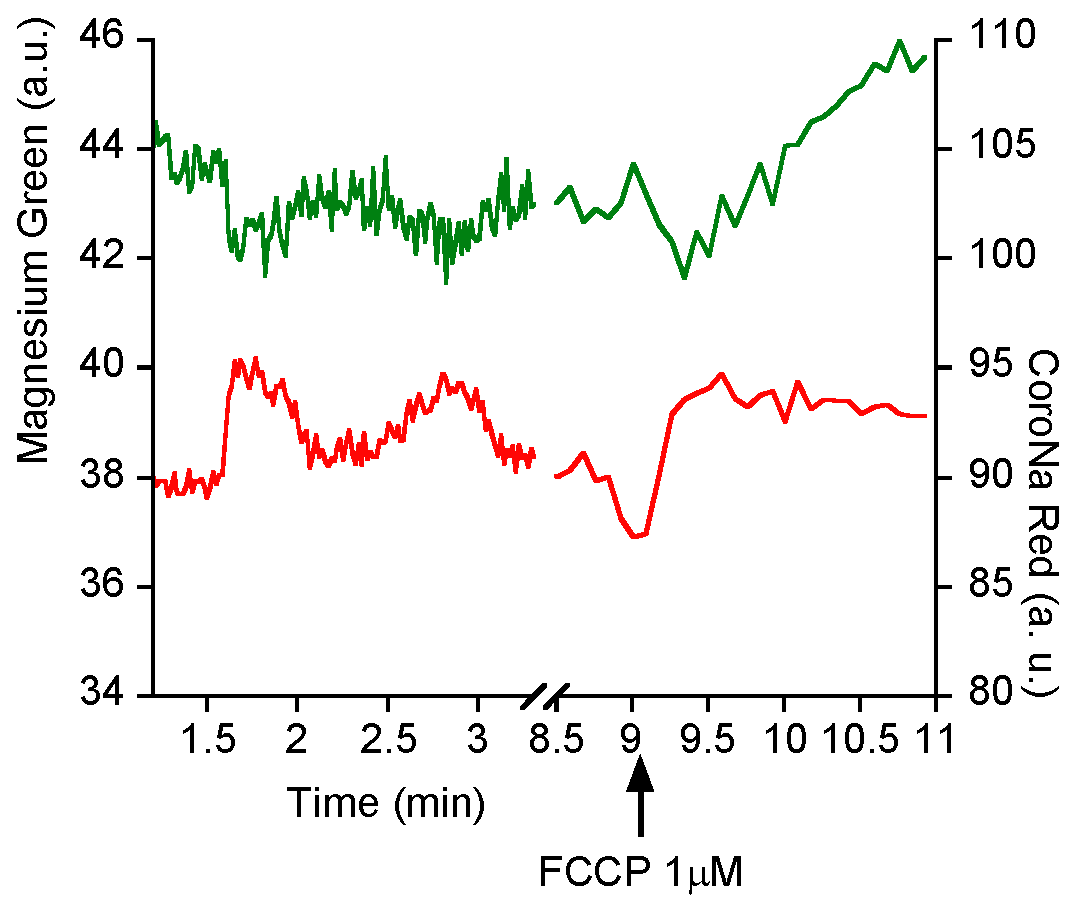

Supplement: Figure S5 — Mitochondrial Na+ transients are coincident with transient decrease in cytosolic free Mg2+ concentration. Astrocytes were simultaneously loaded with Magnesium Green and CoroNa Red to monitor the Mg2+ concentration and mitochondrial Na+ concentration, respectively. As a control, the mitochondrial uncoupler FCCP (10 µM, 30 µL) was added to the 270 µL of buffer at the end of each experiment. Representative trace of 6 experiments (24 mitochondria) and 5 experiments (19 mitochondria) under widefield and TIRF microscopy, respectively. (TIF) [file pone.0028505.s005.tif]
